# Variation in hypertension clinical practice guidelines: a global comparison

**DOI:** 10.1186/s12916-021-01963-0

**Published:** 2021-05-12

**Authors:** Richu Philip, Thomas Beaney, Nick Appelbaum, Carmen Rodriguez Gonzalvez, Charlotte Koldeweij, Amelia Kataria Golestaneh, Neil Poulter, Jonathan M. Clarke

**Affiliations:** 1 Department of Primary Care and Public Health, Imperial College London, London, UK; 2 Helix Centre for Design in Healthcare, Institute of Global Health Innovation, Imperial College London, London, UK; 3 Imperial Clinical Trials Unit, Imperial College London, London, UK; 4 Centre for Mathematics of Precision Healthcare, Department of Mathematics, Imperial College London, South Kensington Campus, London, SW7 2AZ UK

## Abstract

**Background:**

Hypertension is the largest single contributor to the global burden of disease, affecting an estimated 1.39 billion people worldwide. Clinical practice guidelines (CPGs) can aid in the effective management of this common condition, however, inconsistencies exist between CPGs, and the extent of this is unknown. Understanding the differences in CPG recommendations across income settings may provide an important means of understanding some of the global variations in clinical outcomes related to hypertension.

**Aims:**

This study aims to analyse the variation between hypertension CPGs globally. It aims to assess the variation in three areas: diagnostic threshold and staging, treatment and target blood pressure (BP) recommendations in hypertension.

**Methods:**

A search was conducted on the MEDLINE repository to identify national and international hypertension CPGs from 2010 to May 2020. An additional country-specific grey-literature search was conducted for all countries and territories of the world as identified by the World Bank. Data describing the diagnosis, staging, treatment and target blood pressure were extracted from CPGs, and variations between CPGs for these domains were analysed.

**Results:**

Forty-eight CPGs from across all World Bank income settings were selected for analysis. Ninety-six per cent of guidelines defined hypertension as a clinic-based BP of ≥140/90 mmHg, and 87% of guidelines recommended a target BP of < 140/90 mmHg. In the pharmacological treatment of hypertension, eight different first-step, 17 different second-step and six different third-step drug recommendations were observed. Low-income countries preferentially recommended diuretics (63%) in the first-step treatment, whilst high-income countries offered more choice between antihypertensive classes. Forty-four per cent of guidelines, of which 71% were from higher-income contexts recommended initiating treatment with dual-drug therapy at BP 160/100 mmHg or higher.

**Conclusion:**

This study found that CPGs remained largely consistent in the definition, staging and target BP recommendations for hypertension. Extensive variation was observed in treatment recommendations, particularly for second-line therapy. Variation existed between income settings; low-income countries prescribed cheaper drugs, offered less clinician choice in medications and initiated dual therapy at later stages than higher-income countries. Future research exploring the underlying drivers of this variation may improve outcomes for hypertensive patients across clinical contexts.

## Introduction

Hypertension is the largest single contributor to the global burden of disease, affecting an estimated 1.39 billion people worldwide and accounting for 10.4 million premature deaths per year [1, 2]. Despite the trajectory suggesting a continuing increase in hypertension prevalence globally, there are large numbers of undiagnosed and inadequately controlled hypertensive patients [3]. A 2017 multinational cross-sectional study found that 35% of individuals had hypertension, of whom 58% were receiving antihypertensive treatment, and of those on treatment, 46% did not achieve adequate blood pressure (BP) control [3].

Disparities in hypertension prevalence, awareness, management and control exist between country income settings. The age-standardised prevalence of hypertension fell by 2.6% from 2000 to 2010 in high-income countries (HICs), and rose by 7.7% in low- and middle-income countries (LMICs) over the same period [2]. As of 2015, the majority of hypertensive patients live in LMICs [4]. Additionally, awareness, treatment and control are increasing at slower rates in LMIC settings than in HICs [2].

Adequate management of hypertension improves outcomes from several major health conditions. A 2017 meta-analysis found that a 10-mmHg decrease in systolic blood pressure (SBP) significantly reduces the risk of major CVD events, coronary heart disease, stroke and heart failure, decreasing all-cause mortality by 13% in the study population [5]. Achieving adequate BP control is particularly important as hypertension and associated conditions are responsible for significant economic costs. In 2013, the combined direct (treating hypertension) and indirect (associated comorbidities) costs of managing hypertension were $51.2 billion in the USA alone [6]. It has been estimated that the costs of complications due to hypertension outweigh the cost of managing hypertension itself, indicating that effective hypertension management may have wide-reaching economic benefits to health systems [7, 8].

Efforts to improve the quality of care for patients with hypertension have, in part, involved the translation of available evidence on the effectiveness of current treatments into guidance documents for clinicians. Recent years have seen the widespread development and dissemination of clinical practice guidelines (CPG) by learned bodies, international societies and local care providers [9]. A CPG is defined by the Institute of Medicine as a “systematically developed statement that aids with clinician and patient decisions regarding specific clinical conditions” [10]. CPGs emerged as a means to standardise medical practice, ensure cost-effectiveness and enhance patient care [9].

Currently, there are many local, national and international guidelines produced by different organisations that give recommendations for the management of hypertension. Studies comparing these guidelines have shown variation in recommendations for the diagnosis, treatment and treatment targets for patients with hypertension [11–13]. However, these studies compared a small number of guidelines, mainly from HICs and offered only a brief insight into their similarities and differences. Hence, scope exists to compare hypertension guidelines on a far larger scale and, importantly, across income settings.

As a condition for which the affected population predominantly live in LMICs, knowledge of variation in recommendations made to clinicians treating patients with hypertension in these settings remains poorly understood. This study aims to address this deficiency by examining the extent of variation across clinical practice guidelines for the management of hypertension internationally. The primary aim is to determine whether variation exists between CPGs for the management of hypertension through analysis of national and international guidelines from different income settings. Specifically, this study aims to compare the following:

- Diagnostic thresholds for hypertension and staging of hypertension
- Recommended treatment strategies for uncomplicated primary hypertension (in the absence of comorbidities)
- Target BP for patients with uncomplicated primary hypertension
- Whether different targets are recommended for the elderly and other at-risk subpopulations

## Methods

### Search strategy

Using the MEDLINE repository, the terms “(blood pressure OR hypertension) AND (guideline*)” were used to conduct a semi-systematic search to identify CPGs. All papers that included these terms in the titles and written in English were screened for relevance. To ensure the inclusion of a variety of global and international guidelines, an additional search of grey literature was conducted using the Google search engine. To ensure consistency, identical search terms as above were used, with the addition of specific countries: “(blood pressure OR hypertension) AND (guideline) AND ([COUNTRY NAME])”. This strategy was used to identify hypertension CPGs, where present, from each of the 196 individual countries and territories recognised by the World Bank [14]. The first ten search results returned from Google for each country were examined for relevance. The aim of this search strategy was not to be exhaustive but to ensure the inclusion of a wide range of CPGs from varying settings.

### Inclusion criteria

National and international guidelines that comprised statements on the management of hypertension, written in English, published between January 2010 and May 2020 were included. A document was deemed a guideline if it explicitly identified itself as a guide for clinical decision-making. Titles and abstracts matching these criteria were screened for relevance and included if they contained specific recommendations on the pharmacological treatment of hypertension. If multiple hypertension-specific guidelines were identified produced by different governing bodies from the same country, all were selected, providing the inclusion criteria were met. Where more than one published guideline by the same governing body was found, the most recent guideline was selected for analysis. To capture hypertension guidance for countries where no specific hypertension guideline had been published, guidelines that provided guidance on multiple conditions were included, provided they contained recommendations for the treatment of hypertension.

### Data extraction

Guidelines were stratified by country income, based on the World Bank definitions into low-income countries (LICs), lower-middle-income countries (lower-MICs), upper-middle-income countries (upper-MICs) and HICs [14]. Data pertaining to hypertension diagnosis, treatment and treatment targets were extracted from each guideline and recorded using Microsoft Excel. Data were collected across three domains of diagnosis, treatment and treatment targets as follows:

1. Diagnosis:
  - BP threshold for diagnosis (thresholds based on office-based BP readings were used, as these were likely to be the most commonly available and feasible method for diagnosis across all resource settings)
  - Staging of hypertension
2. Treatment:
  - Recommendation of non-pharmacological methods
  - Threshold BP for initiating pharmacological therapy
  - First-step, second-step and where available third-step drug therapy for hypertension
  - The recommendation of initiation with monotherapy or dual therapy
3. Treatment targets for specific patient groups:
  - Patients with uncomplicated primary hypertension
  - The elderly
  - Patients with comorbidities as outlined by the CPGs

### Characterising variation

Data were analysed for variation for each of the aforementioned parameters. Differences in the diagnosis and treatment of hypertension between guidelines were described and quantified. The most commonly used first-step, second-step and third-step drug therapies overall and across World Bank income settings were identified. Pharmacological treatment pathways were represented using Sankey diagrams across all guidelines and according to national income levels. All figures were produced using python version 3.6.8 with the plotly (version 4.14.3), geopandas (version 0.8.0) and matplotlib (version 3.3.2) libraries.

## Results

**Fig. 1:**
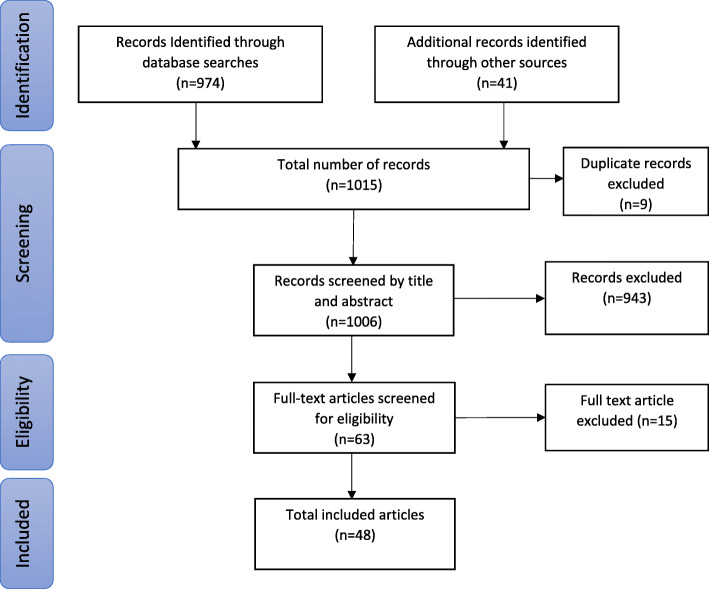
PRISMA diagram detailing search results

**Fig. 2:**
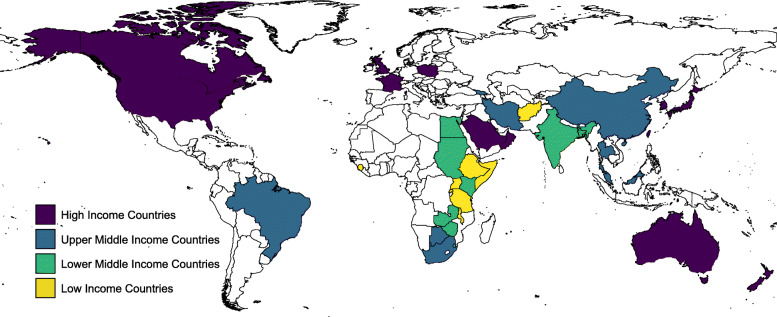
World map showing the location and World Bank income levels of countries whose guidelines were included in the study. Kiribati, Hong Kong and Fiji are not shown due to their size

The search strategy returned 974 records from MEDLINE; 17 of which met the inclusion criteria for selection. A further 31 guidelines were found through country-specific searches on Google. Details of search results are presented in Fig. 1, and the complete list and income classification of guidelines are found in Additional File 1. Forty-eight guidelines were included in the study, from 45 countries and territories. Eight (17%) were from LICs [15–22], 10 (21%) from lower-MICs [23–32], 11 (23%) from upper-MICs [33–44], 17 (35%) were from HICs [45–63] and 2 (4%) catered for varied income settings [64, 65]. The geographic location and income levels of included countries are shown in Fig. 2.

### Diagnostic threshold

Ninety-six per cent (*n* = 46) of guidelines provided an explicit numerical diagnostic threshold for hypertension, of which 93% (*n* = 43) included distinct stages for classifying hypertension. The Hypertension Canada 2020 [45], the French Society of Hypertension 2013 [65] and the 2017 Essential Guidelines of Tanzania [20] provided diagnostic thresholds but did not stage hypertension. Hypertension was defined as a clinic-based BP ≥140/90 mmHg by 96% (*n* = 44) of guidelines. Two definitions that varied from the above were the 2017 American College of Cardiology/American Heart Association 2017 (ACC/AHA 2017) guideline [55] (≥130/80 mmHg) and the 2014 Egyptian Hypertension Society (EHS) guidelines [30] (≥150/95 mmHg). Of the 43 CPGs that staged hypertension, 77% (*n* = 33) utilised a 3-stage classification whilst 23% (*n* = 10) divided it into two stages.

The 2017 ACC/AHA guideline’s recommendation to lower the diagnostic threshold to ≥130/80 mmHg was in part influenced by the Systolic Blood Pressure Intervention Trial (SPRINT) [66]. Fifty-six per cent (*n* = 27) of guidelines that were identified by our study were published after the publication of SPRINT, i.e. from 2016 onwards. Fifty-two per cent (*n* = 14) of these guidelines discussed outcomes from SPRINT and an additional 7% (*n* = 2) cited the trial without further discussion, but the ACC/AHA guidelines were the only ones found to have lowered the diagnostic threshold influenced by SPRINT. Sixty-four per cent (*n* = 9) of the guidelines which discussed SPRINT were from HICs, and the remaining 36% (*n* = 5) were from lower- or upper-MICs. No LIC guidelines published after 2016 discussed or cited SPRINT.

### Treatment

Ninety-eight per cent (*n* = 47) of guidelines gave explicit recommendations on non-pharmacological lifestyle interventions in the management of hypertension. The 2013 Botswanan primary care guideline [38] was the only guideline that did not explicitly mention lifestyle factors.

Sixty-nine per cent (*n* = 33) of guidelines advised direct initiation of antihypertensives at a BP of ≥160/100 mmHg, without a trial period of lifestyle interventions alone. Twelve per cent (*n* = 6) advised direct treatment at a BP of ≥140/90 mmHg, and 7% (*n* = 3) recommended direct pharmacological intervention at a BP of ≥180/110 mmHg. The remaining 12% (*n* = 6) did not make their advice explicitly clear. All guidelines which recommended initiating direct drug therapy at a BP of ≥140/90 mmHg were from higher-income settings.

**Fig. 3:**
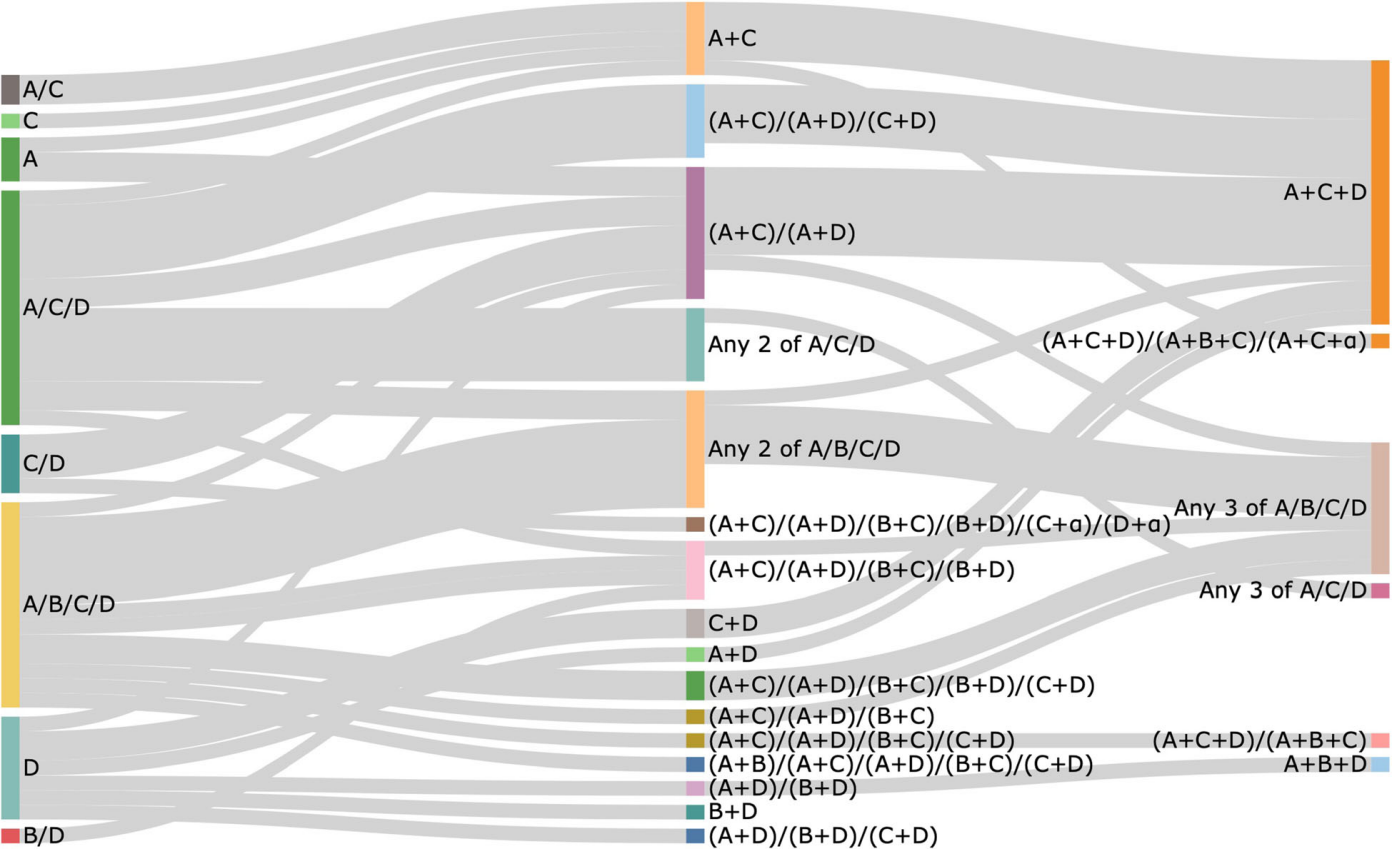
Sankey diagram illustrating the 1st-, 2nd- and 3rd-step drug recommendations made by the 48 guidelines. A, ACEi or ARB; B, beta-blocker; C, CCB; D, diuretic; α, alpha-blocker. “/” indicates a choice between drug classes, whilst “+” indicates concurrent prescription of multiple drug classes. The size of pathways is representative of the number of guidelines making recommendations

**Fig. 4:**
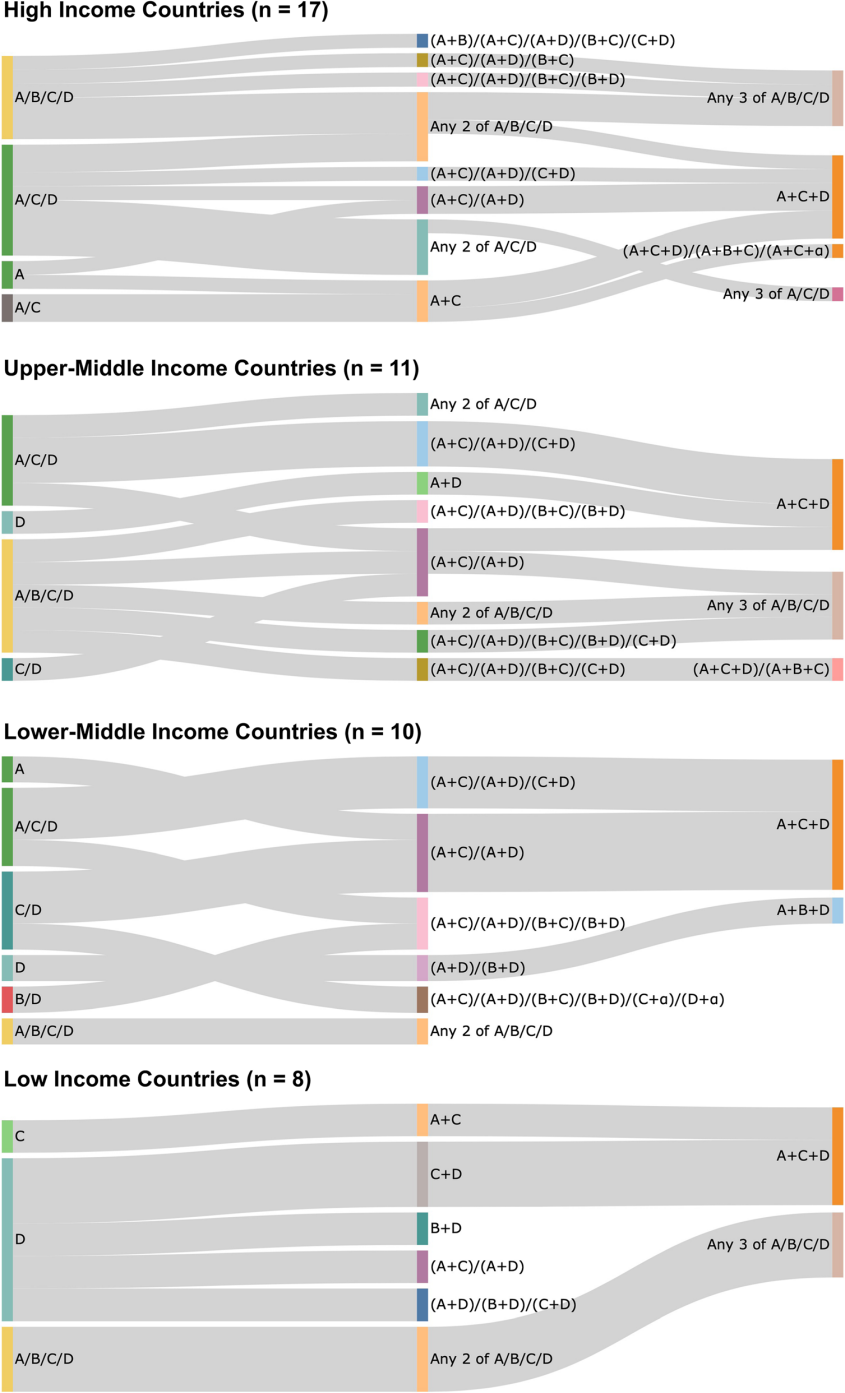
Sankey diagrams illustrating the 1st-, 2nd- and 3rd-step therapy recommendations made by different income settings. A, ACEi or ARB; B, beta-blocker; C, CCB; D, diuretic; α, alpha-blocker. “/” indicates a choice between drug classes, whilst “+” indicates concurrent prescription of multiple drug classes

The order and combination in which these drugs were recommended by the 48 CPGs at first-step, second-step and third-step are illustrated in Fig. 3. There were 8 different first-step recommendations, 17 different second-step combinations and 6 different third-step combinations seen between the 48 guidelines. Variations in recommended pharmacological therapy were observed between income settings, as illustrated by the Sankey diagrams in Fig. 4. Table 1 summarises the most common first-, second- and third-step drug therapies recommended by all guidelines and between income settings.

**Table 1:** Commonly recommended drug therapies by income setting

Income setting	First-step therapy	Second-step therapy	Third-step therapy
All (*n* = 48)	Any one of: • ACEi/ARB • CCB • Diuretic (33% of CPGs)	ACEi + CCB/diuretic (19% of CPGs)	ACEi/ARB + CCB + Diuretic (59% of CPGs)
Low-income countries (*n* = 8)	Diuretic (63% of CPGs)	No distinct majority (6 different combinations)	ACEi/ARB + CCB + Diuretic (60% of CPGs)
Lower-middle income countries (*n* = 10)	CCB/diuretic (30% of CPGs)	CCB/diuretic + ACEi/ARB (30% of CPGs)	ACEi/ARB + CCB + Diuretic (83% of CPGs)
Upper-middle income countries (*n* = 11)	Any one of: • ACEi/ARB • CCB • Diuretic • Beta-blocker (45% of CPGs)	No distinct majority (7 different combinations)	ACEi/ARB + CCB + Diuretic (50% of CPGs)
High-income countries (*n* = 18*) *Including ISH 2020 optimal	Any one of: • ACEi/ARB • CCB • Diuretic (47% of CPGs)	No distinct majority (8 different combinations)	ACEi/ARB + CCB + Diuretic (50% of CPGs)

The most common first-, second- and third-step drug therapy recommended by all guidelines and by differing income settings, including the percentage of guidelines which made the recommendations

Across the selected guidelines, the most common classes of antihypertensive medication recommended were angiotensin-converting enzyme inhibitors (ACEi), angiotensin receptor blockers (ARB), beta-blockers, calcium channel blockers (CCB) and diuretics. Only two guidelines recommended alpha-blockers (Brunei 2019 and Zimbabwe 2015) [32, 53], and other antihypertensives such as potassium-sparing diuretics were not observed in the first three steps. Of the 47 guidelines that recommended either an ACEi or an ARB, 68% (*n* = 32) did not preferentially recommend one over the other. In light of this, ACEi/ARB were combined as one category of medication in subsequent analysis. The remaining 32% of guidelines (*n* = 15) recommended ACEi in preference to ARBs in patients with uncomplicated hypertension, with some recommending ARBs if ACEi are not tolerated due to side effects. Sixty per cent (*n* = 9) of the 15 guidelines which preferentially recommended ACEi were from LICs and lower-MICs.

Regarding diuretics, 44% (*n* = 21) recommended thiazide diuretics, 8% (*n* = 4) recommended thiazide-like diuretics and 40% (*n* = 19) offered a choice between either thiazide or thiazide-like diuretics. Eight per cent (*n* = 4) referred to “thiazide-type” diuretic, without providing clarification of the precise subgroup. Thiazide and thiazide-like diuretics were therefore combined into a single category. At all steps, 57% of CPGs that recommended thiazide diuretics, as opposed to thiazide-like diuretics, were from LICs or lower-MICs. In addition, the 2020 International Society of Hypertension (ISH) [65] included “essential” and “optimal” recommendations to account for low- and high-resource settings. These advised lower-resource settings to use any antihypertensive based on availability and ideal characteristics.

For patients with a BP ≥160/100 mmHg, initiation with monotherapy was recommended by 33% (*n* = 16) of guidelines. Forty-four per cent (*n* = 21) recommended direct initiation with dual-drug therapy, of which 71% (*n* = 15) were from higher-income settings. Ten per cent (*n* = 5) of CPGs gave a choice to initiate treatment with either monotherapy or dual therapy, and the remaining 13% (*n* = 6) did not make clear recommendations.

### Target blood pressure

For uncomplicated hypertension, 96% (*n* = 46) of guidelines provided data on target BPs. For patients with uncomplicated hypertension, 87% (*n* = 40) of guidelines recommended a target BP < 140/90 mmHg, and 9% (*n* = 4) recommended a target BP of < 130/80 mmHg. The 2014 Kiribati CPG had a target BP of systolic BP < 160 mmHg, and the 2014 Egyptian CPG recommended a target BP < 150/95 mmHg. Eighty-eight per cent of CPGs had a target BP that was identical to their diagnostic threshold for hypertension.

For the elderly, 46% (*n* = 22) of guidelines recommended an alternative target blood pressure. The definition of “elderly” varied between guidelines (ages: *n* = 8 ≥ 80, *n* = 7 ≥ 60, *n* = 4 ≥ 65, *n* = 2 ≥ 75 and *n* = 1 ≥ 55), and three guidelines divided the elderly population into “elderly” and “very elderly”. The most common target BP for elderly patients, recommended by 45% (*n* = 10) of the guidelines, was < 150/90 mmHg. Guidelines which differed from this BP target are summarised in Table 2.

**Table 2:** Variation in target blood pressure for elderly patients

Guideline	Recommended target BP in the elderly
Poland 2017 [58] Somalia 2015 [21]	SBP 140–150 mmHg
Thailand 2015 [39]	Age 60–80, 140–150/90 mmHg Age ≥ 80, < 150/90 mmHg
China 2019 [44]	Age 65–80, < 140/90 mmHg Age ≥ 80, < 150/90 mmHg
IGH India 2013 [27]	Age 55–79, < 140/90 mmHg Age > 80, SBP 140–140 mmHg
ISH 2020 [65] JSH/Japan 2019 [54]	< 140/90 mmHg
Korea 2018 [52]	SBP < 140 mmHg
ESC/ESH 2018 [56]	SBP 130–139 mmHg DBP 70–79 mmHg
Taiwan 2017 [60]	SBP < 140/90
ACC/AHA 2017 [55]	Age ≥ 65, SBP < 130 mmHg

Target BP for elderly patients recommended by CPGs that differed from the most common target of < 150/90 mmHg

*IGH* Indian Guidelines for Hypertension, *JSH* Japanese Society of Hypertension, *ESC/ESH* European Society of Cardiology and Hypertension

Fifty-four per cent (*n* = 26) of guidelines specified different target BPs for subpopulations with comorbidities; some of the common comorbidities included were those with diabetes, chronic kidney disease and cardiovascular disease. This study did not assess the specific recommendations for each of these subgroups; however, guidelines most commonly recommended a lower target BP of < 130/80 mmHg for populations they deemed to be at a higher risk (85%, *n* = 22).

## Discussion

This study found that CPGs for the management of hypertension exist across all income settings; however, more guidelines were found from upper-MICs and HIC settings (61%). Moreover, of those found through our search strategy, 100% of CPGs from LICs provided guidance on multiple conditions, whereas all CPGs from HICs were specific to hypertension, suggesting a current gap in specific hypertension CPGs in LICs. This is supported by a 2016 systematic review which found that fewer hypertension guidelines were produced in LICs and lower-MICs [67]. Analysis from this study found consensus in the diagnostic thresholds and target BPs for uncomplicated hypertension but observed extensive variation in the treatment strategies recommended.

Consensus was observed between guidelines in the diagnostic threshold and staging of hypertension. Ninety-five per cent of guidelines defined hypertension as an SBP of ≥140 mmHg or a DBP of 90 mmHg. However, the latest 2017 ACC/AHA guidelines lowered this widely accepted threshold to ≥130/80 mmHg [55], influenced in part by the 2015 SPRINT, which found lower rates of cardiovascular events in those treated to lower BP targets [66]. Using a lower threshold for defining hypertension worldwide would greatly increase its prevalence, resulting in more patients becoming eligible for pharmacological therapy, thereby placing an increased burden on health systems to treat and monitor hypertension [68, 69]. Since the publication of SPRINT, no guideline other than the ACC/AHA 2017 guidelines [55], has lowered the diagnostic threshold for hypertension, despite the findings that 52% of guidelines published from 2016 onwards discussed the findings of SPRINT. It remains to be seen whether other guidelines will follow the ACC/AHA in lowering the diagnostic threshold.

Consensus was also found between guidelines on the inclusion of recommendations of lifestyle interventions to manage hypertension, being advised in 98% of guidelines. This guidance is in line with extensive evidence of the importance of lifestyle modifications, such as dietary changes and exercise in reducing BP [70–72]. Guidelines also remained somewhat consistent in recommending that drug therapy should be initiated, without a trial period of non-pharmacological interventions in cases where blood pressure is 160/100 mmHg or higher.

Four broad classifications of antihypertensives, ACEi/ARBs, CCB, beta-blocker and diuretics, were most commonly used by the 48 guidelines examined but in very different sequences and combinations. The greatest level of variation was observed within second-step therapy, where 17 different combinations of pairs of drugs were recommended. In the third-step therapy, the extent of variation was reduced, with only six combinations. Evidence suggests that the effectiveness of ACEi/ARBs, CCBs and diuretics (and to a lesser extent, beta-blockers) is largely similar in lowering BP [73, 74]. This could explain the variation seen, with no strong preference amongst the different guidelines in second-step therapy. Country-specific contexts and patient-specific factors may also influence CPGs to recommend differing therapies according to their populations. For instance, the 2019 National Institute for Health and Care Excellence (NICE) guidelines [49] suggest different management strategies by ethnicity; however, there is a need for further robust studies to determine whether antihypertensive treatment outcomes vary in different ethnic populations [75].

Differences were apparent across country income settings, with an increased level of clinician choice in drug therapy options with increasing affluence. For instance, in the first-step, there was a trend towards LICs recommending diuretics, lower-MICs offering a choice between a diuretic and a CCB and upper-MICs and HICs advising any one of an ACEi, ARB, CCB and diuretic. A systematic review found that allowing clinicians’ greater autonomy in choice of management in a CPG can encourage adherence to guidelines. However, this review was primarily based on studies of physicians from HICs and so may not be generalisable to other healthcare workers and income contexts [76]. The benefits of restrictive or permissive guidelines across clinical contexts and guideline users remain an important and as yet unanswered question.

Greater flexibility in the choice of drugs may reflect the cost and accessibility of BP therapies in different contexts. There was a clear preference for diuretics and CCBs seen amongst LICs and lower-MICs for first- and second-step therapy. Diuretics are one of the least expensive antihypertensive classes [77]; based on the British National Formulary prices, thiazide diuretics (bendroflumethiazide) are the least expensive at £0.33 per pack, followed by CCBs (amlodipine) at £0.46, beta-blockers (atenolol) at £0.63 and thiazide-like diuretics (indapamide) at £0.70. In comparison, ACEi (ramipril) are priced at £2.10 and ARBs (losartan) at £5.09 [78]. At a population level, these translate to large differences in cost and could explain the preferential recommendation of diuretics and CCBs in lower-income contexts, as well as the preference of ACEi to ARBs.

CPGs from higher-income contexts were more likely to recommend initiating dual-drug therapy at a BP ≥160/100 mmHg. Recent evidence suggests that most patients will require two drug classes in order to achieve adequate BP control [79, 80]. CPGs from lower-income settings may have opted for the initiation with monotherapy again due to barriers including the low availability of affordable drugs [2]. Studies have shown that low- and middle-income countries often closely follow the release of guidelines from high-income regions [67], for instance, the 2016 Government of India Hypertension CPG [25] from this study, explicitly states that the guideline is “adopted and/or adapted from existing evidence-based guidelines”, including the European Society of Cardiology/European Society of Hypertension (ESC/ESH), Joint National Committee (JNC) and NICE guidelines [57, 81, 82]. The Indian guidelines made it clear that they chose aspects from each of these guidelines which were best suited to their particular context. Changes such as the first-line use of combination therapy as recommended by HIC guidelines may not be feasible in low-resource regions. Recognising these difficulties, the ISH 2020 [65] guidelines have produced “essential” recommendations for lower-income settings, which advocate the use of any available antihypertensives, compared to “optimum” recommendations, in order to act as a global resource; this was the only guideline assessed by this study to use this approach.

For patients that were hypertensive, most CPGs recommended a target BP of less than 140/90 mmHg whilst 9% of guidelines recommended a target of < 130/80 mmHg. The ESC/ESH 2018 guidelines recommended lower targets of SBP between 120 and 129 mmHg and a DBP between 70 and 79 mmHg in patients under 65 years [56]. The lower BP targets recommended by these guidelines are partly influenced by findings from SPRINT, where lower rates of fatal and non-fatal cardiovascular events and deaths were observed in cohorts treated to a target systolic BP of less than 120 mmHg, compared to less than 140 mmHg [66]. However, the methodology used in BP measurement may not be applicable to routine clinical practice [83], and following the publication of SPRINT, a Cochrane review updated in 2017, 2018 and 2020 based on six different randomised controlled trials (RCTs) showed no reduction in total mortality or serious adverse events when treating to a target less than 135/85 mmHg [84–86]. The current mixed and evolving evidence regarding optimal BP targets offers a potential reason behind the variations in targets recommended by different CPGs. It is of note that most CPGs (88%) recommended a target BP for hypertensive patients that was identical to their diagnostic threshold for hypertension.

Many CPGs recommended different management strategies for elderly patients, with most opting for a higher BP target of < 150/90 mmHg. Higher targets may result from a trade-off between achieving adequate BP control and minimising adverse drug effects, which are more common in elderly patients [87]. There was a significant variation in the definition of the term “elderly”, ranging from age ≥ 55 to ≥80 years between CPGs. No clear correlation to income settings was seen between the age definitions chosen by guidelines. It may partly be attributed to variation in life expectancy across the countries from which CPGs were identified, and some CPGs explicitly mention their populations’ life expectancy when determining the cut-off age [60]. In addition to differing thresholds for the elderly, many guidelines also included specific thresholds for comorbidities, including diabetes, chronic kidney disease and cardiovascular disease. There was a general consensus towards lower target BPs in these groups (BP < 130/80 mmHg) which could reflect the need for more stringent BP control in these high-risk patients [88].

Our study highlights extensive international variation in guidelines for the management of hypertension both within and between income levels. Variation within countries was also observed, in the case of the USA with the AHA/ACC 2017 and JNC 2014 offering different diagnostic thresholds and target BP recommendations [55, 57]. As CPGs aim to be implementable digests of the best available evidence, the observed variation may represent the complex conflict between currently available evidence and what may be achieved at scale in local contexts. Additionally, what is known about the treatment of hypertension is ever-changing with the emergence of new scientific studies that may conflict with or support existing literature. A degree of dissensus between guidelines is therefore expected where the underlying evidence base for a condition is itself in flux.

The clinical guidelines featured in this study are expected to support clinicians’ decision-making across all aspects of care from the diagnosis of a condition to its treatment and monitoring. Whilst evidence from robust clinical studies may support certain crucial aspects of this process, other areas may be relatively neglected by academic scrutiny. A study analysing the evidence base of ACC/AHA 2008 guidelines found that only 11% of recommendations were made based on very strong evidence, whereas a median of 48% of recommendations was based on weaker evidence. This finding suggests that the production of a clinical guideline for the treatment of hypertension cannot be solely reliant on robust clinical evidence and that expert opinion and organisational preference may play a role in producing guidelines [89]. Similarly, the dominance of hypertension research from high-income settings results in new evidence answering questions of particular relevance to a minority of the global hypertensive population, using treatment strategies that may not be applicable to the local logistical and financial constraints of the global majority. Guidelines especially in lower-income settings may focus on what is most feasible for the particular resource setting as opposed to the optimal.

The inclusion of guidelines that are written only in English led to the exclusion of data from non-English-speaking settings. In addition, as the search strategy did not exhaustively examine all online content, eligible guidelines may have been missed by the search, particularly guidelines that encompass recommendations for multiple conditions which may not have included the terms “hypertension” or “blood pressure” in their titles or those that did not identify themselves as a guideline. Guidelines from some settings may have only been available as hard copies and would have been missed in the search strategy. As a result, the methodology may have been biassed towards including CPGs from higher-income settings. However, the aim was not to exhaustively identify all guidelines, but to provide relative comparisons between guidelines in a range of different income settings, which were all represented. In addition, this study did not seek to explore the quality and evidence base behind each CPG or to identify the extent to which these guidelines are followed in their local contexts, which were outside of the scope of the present study. Our analysis also focusses on the main thresholds and treatment presented in guidelines, which may differ for specific patient groups and in the presence of other co-morbid conditions, such as diabetes.

This study objectively characterised the variation between hypertension guidelines globally, but further research is needed to explore the underlying reasons for the divergence seen between CPGs. The evidence base used in the specific recommendations in CPGs will vary, highlighted by our findings that only half of the guidelines published in the year following the SPRINT trial included reference to its findings, and further research exploring the difference in the evidence base used in guideline creation is needed. Further qualitative research should also be conducted into the creation, utilisation and perception of hypertension CPGs by healthcare practitioners across a range of income settings, and how local context influences recommendations. In addition, whilst this study outlines variations in hypertension guidelines on a national and international level, there is further scope to assess the variation at a more local scale, within countries.

## Conclusion

This study identified 48 national and international guidelines for the management of hypertension. BP thresholds for the diagnosis and staging of hypertension, as well as BP target recommendations, were largely consistent across guidelines and across income settings. However, recommendations on antihypertensive drug therapy at each treatment step differed greatly, with guidelines from higher-income settings offering greater clinician autonomy in choice of antihypertensive drugs and dual-combination therapies, in contrast to lower-income settings, which may reflect drug costs or availability. The variation seen may represent the lack of a robust evidence base on management, particularly for lower-income settings, given research is focused on higher-income countries. Further research is needed to explore the reasons for such divergence in guidelines to better inform those involved in their creation and the clinicians using them.

## Supplementary Information

**Additional file 1.** : List of all guidelines included in the study, including full title, country of origin, year of publication and the correlating reference number as per manuscript.

## Data Availability

The datasets used and/or analysed during the current study are available from the corresponding author on reasonable request.
