# Supplementary material for: Variation in hypertension clinical practice guidelines: a global comparison
Source: BMC Med. 2021 May 12;19:117. doi: 10.1186/s12916-021-01963-0 (PMC8114719; doi:10.1186/s12916-021-01963-0)
Supplement: Supplementary file 1 — Additional file 1. : List of all guidelines included in the study, including full title, country of origin, year of publication and the correlating reference number as per manuscript. [file 12916_2021_1963_MOESM1_ESM.docx]

**Additional File 1**

List of all guidelines included in the study, including full title, country of origin, year of publication and the correlating reference number as per manuscript.

| Income setting | Guideline | Country | Year | Ref |
| --- | --- | --- | --- | --- |
| LICs | National Standard Treatment Guidelines for the Primary Level | Afghanistan | 2013 | 15 |
|  | Malawi Standard Treatment Guidelines | Malawi | 2015 | 16 |
|  | Ministry of Health Non-Communicable Diseases: Diagnosis and Treatment Guide | Sierra Leone | 2017 | 17 |
|  | Ugandan Clinical Guidelines: National Guidelines for the Management of Common Conditions | Uganda | 2012 | 18 |
|  | National Guidelines for the management of NCDs | Rwanda | 2016 | 19 |
|  | Standard Treatment Guidelines and National Essential Medicines list | Tanzania | 2017 | 20 |
|  | Hospital and Referral Health Centre Guidelines | Somalia | 2015 | 21 |
|  | Guidelines on Clinical and Programmatic Management of Major NCDs | Ethiopia | 2016 | 22 |
| Lower-MICs | National Guidelines for the management of hypertension | Bangladesh | 2013 | 23 |
|  | Kenya National Guidelines for Cardiovascular Disease Management | Kenya | 2018 | 24 |
|  | Government of India- Standard Treatment Guidelines for Hypertension | India | 2016 | 25 |
|  | Sudan Hypertension Guideline | Sudan | 2012 | 26 |
|  | Indian Guidelines on Hypertension | India | 2013 | 27 |
|  | Standard Treatment Guidelines and Essential Medicines list | Zambia | 2017 | 28 |
|  | Therapeutic Drug Guidelines Kiribati Ministry of Health: Book 2, Cardiovascular | Kiribati | 2014 | 29 |
|  | The Egyptian Hypertension Society Guidelines | Egypt | 2014 | 30 |
|  | Standard Treatment Guidelines and Essential Medicines List of Common Medical Conditions in the Kingdom of Swaziland | Swaziland | 2012 | 31 |
|  | 7th Essential Medicines List and Standard Treatment Guidelines for Zimbabwe | Zimbabwe | 2015 | 32 |
| Upper-MICs | Cardiovascular Therapeutic guidelines | Fiji | 2015 | 33 |
|  | South African hypertension practice guideline | South Africa | 2014 | 34 |
|  | Hypertension Management Guide Georgia Department of Public Health | Georgia | 2015 | 35 |
|  | 7th Brazilian Guideline for Arterial Hypertension | Brazil | 2016 | 36 |
|  | Clinical Practice Guidelines Management of Hypertension | Malaysia | 2018 | 37 |
|  | Botswana Primary Care Guidelines for Adults | Botswana | 2013 | 38 |
|  | 2015 Thai Hypertension Guideline | Thailand | 2015 | 39 |
|  | Guidelines for management of hypertension; Ministry of Health Jamaica | Jamaica | 2014 | 40 |
|  | The first Iranian recommendations on prevention, evaluation and management of high blood pressure | Iran | 2012 | 41 |
|  | Guidelines for managing Hypertension and related comorbidities in Latin America | Latin America | 2017 | 42 |
|  | 2018 Chinese Guidelines for Prevention and Treatment of Hypertension  2019 Chinese guideline for the management of hypertension in the elderly | China | 2018/  2019 | 43/  44 |
| HICs | Hypertension Canada’s Comprehensive Guidelines for Hypertension | Canada | 2020 | 45 |
|  | Cardiovascular Disease Risk Assessment and Management for Primary Care | New Zealand | 2018 | 46 |
|  | Saudi Hypertension Guidelines; Saudi Hypertension Management Society | Saudi Arabia | 2018 | 47 |
|  | Guideline for the diagnosis and management of hypertension in adults; Australian Heart Foundation | Australia | 2016 | 48 |
|  | NICE guidelines for the management of hypertension | UK | 2019 | 49 |
|  | 2015 Oman Heart Association Guidelines for the Management of Hypertension | Oman | 2015 | 50 |
|  | Ministry of Health Clinical Practice Guidelines: Hypertension | Singapore | 2018 | 51 |
|  | 2018 Korean Society of Hypertension Guidelines for the management of hypertension | Korea | 2018 | 52 |
|  | Brunei Darussalam National Hypertension Guideline | Brunei | 2019 | 53 |
|  | The Japanese Society of Hypertension Guidelines for the Management of Hypertension (JSH 2019) | Japan | 2019 | 54 |
|  | American College of Cardiology/American Heart Association Guidelines 2017 (ACC/AHA) | USA | 2017 | 55 |
|  | European Society of Cardiology and European Society of Hypertension g Guidelines (ESC/ESH) | Europe | 2018 | 56 |
|  | Joint National Committee Hypertension Guidelines 8th Edition (JNC 8) | USA | 2014 | 57 |
|  | Management of hypertension in adults: the 2013 French Society of Hypertension guidelines | France | 2013 | 63 |
|  | Polish Forum for Prevention Guidelines on Hypertension: update 2017 | Poland | 2017 | 58 |
|  | The 2017 Focused Update of the Guidelines of the Taiwan Society of Cardiology (TSOC) and the Taiwan Hypertension Society (THS) for the Management of Hypertension | Taiwan | 2015/  2017 | 59/  60 |
|  | Hong Kong Reference Framework for Hypertension Care for Adults in Primary Care Settings  Hong Kong Reference Framework for Hypertension: update on diagnostic threshold and BP targets | Hong Kong | 2018/  2019 | 61/  62 |
| Other | 2017 guidelines for arterial hypertension management in primary health care in Portuguese language countries. | Portuguese Language Countries | 2017 | 64 |
|  | International Society of Hypertension; Global Hypertension Guidelines (Optimal) | Essential and optimal guidelines | 2020 | 65 |
